# Samm50 Promotes Hypertrophy by Regulating Pink1-Dependent Mitophagy Signaling in Neonatal Cardiomyocytes

**DOI:** 10.3389/fcvm.2021.748156

**Published:** 2021-09-22

**Authors:** Ran Xu, Le Kang, Siang Wei, Chunjie Yang, Yuanfeng Fu, Zhiwen Ding, Yunzeng Zou

**Affiliations:** Shanghai Institute of Cardiovascular Diseases, Zhongshan Hospital, Fudan University, Shanghai, China

**Keywords:** cardiac hypertrophy, mitophagy, Samm50, Pink1, heart failure

## Abstract

Pathological cardiac hypertrophy, the adaptive response of the myocardium to various pathological stimuli, is one of the primary predictors and predisposing factors of heart failure. However, its molecular mechanisms underlying pathogenesis remain poorly understood. Here, we studied the function of Samm50 in mitophagy during Ang II-induced cardiomyocyte hypertrophy via lentiviruses mediated knockdown and overexpression of Samm50 protein. We first found that Samm50 is a key positive regulator of cardiac hypertrophy, for western blot and real-time quantitative PCR detection revealed Samm50 was downregulated both in pressure-overload-induced hypertrophic hearts and Ang II-induced cardiomyocyte hypertrophy. Then, Samm50 overexpression exhibits enhanced induction of cardiac hypertrophy marker genes and cell enlargement in primary mouse cardiomyocytes by qPCR and immunofluorescence analysis, respectively. Meanwhile, Samm50 remarkably reduced Ang II-induced autophagy as indicated by decreased mitophagy protein levels and autophagic flux, whereas the opposite phenotype was observed in Samm50 knockdown cardiomyocytes. However, the protective role of Samm50 deficiency against cardiac hypertrophy was abolished by inhibiting mitophagy through Vps34 inhibitor or Pink1 knockdown. Moreover, we further demonstrated that Samm50 interacted with Pink1 and stimulated the accumulation of Parkin on mitochondria to initiate mitophagy by co-immunoprecipitation analysis and immunofluorescence. Thus, these results suggest that Samm50 regulates Pink1-Parkin-mediated mitophagy to promote cardiac hypertrophy, and targeting mitophagy may provide new insights into the treatment of cardiac hypertrophy.

## Introduction

Heart failure, a complex multifactorial syndrome, has now become a worldwide problem reaching epidemic proportions. Cardiac hypertrophy is regarded as the leading cause of heart failure. The onset of cardiac hypertrophy is characterized by a fetal reprogramming of gene expression where adult genes are repressed and fetal genes are activated, resulting in an imbalance between protein synthesis and degradation (1–3). At the molecular level, pathological hypertrophy has been associated with mitogen-activated protein kinase signaling pathways, insulin-like growth factor-I phosphatidylinositol 3-kinase (PI3K)-AKT/protein kinase B mammalian target of rapamycin (mTOR) signaling pathways, calcium signaling pathway, chromatin remodeling and so on (4–8). Although considerable progress has been made in elucidating the molecular mechanism of cardiac hypertrophy, there remain many unknowns.

Over the last decade, many studies have demonstrated that autophagy participates in the pathogenesis of cardiac hypertrophy. For example, cardiac-specific deficiency of Atg5 mice with pressure overload developed cardiac dysfunction and left ventricular dilatation (9). Furthermore, the key inhibitor of the mTOR pathway rapamycin, which is a potent activator of autophagy, has been reported to prevent cardiac hypertrophy (10, 11). Mitophagy, a special autophagy, removes damaged or redundant mitochondria to maintain heart function in response to various stress and heart disease conditions. PTEN-induced kinase1 (Pink1) is a key molecule for mediating mitophagy. Basally, Pink1 is rapidly degraded when it is translocated into mitochondria. However, in any case, if Pink1 is accumulated in the outer membrane of mitochondria (OMM), it would phosphorylate Parkin leading to activating its ligase activity. OMM proteins would be ubiquitinated, and autophagic receptors are recruited. A series of events eventually lead to the mitochondria being delivered to lysosomes (12–18).

All nuclear DNA-encoded mitochondrial proteins must be transported into mitochondria through channels in the OMM. The channel-forming protein Tom40 and voltage-dependent anion channel proteins (VDACs) play key roles (19). The sorting and assembly machinery (SAM) is critical for membrane integration and assembly of Tom40 and VDACs into the mitochondrial outer membrane. SAMM50 sorting and assembly machinery component (Samm50), a member of the SAM complex, contains a β-barrel domain that is conserved in evolution from bacteria to humans (20, 21). Samm50 is considered to be an essential protein present on the outer mitochondrial membrane, and it has been confirmed that it promotes the biogenesis of ß-barrel protein by directly interacting with the TOM complex. Samm50 interact with core proteins of the mitochondrial contact site and cristae organizing system complex to regulate cristae stability (22). Long-term lack of Samm50 influences the protein quantity of all large respiratory complexes of mitochondrial coding subunits, as well as mitochondrial swollen and mitochondrial inheritance impaired (23, 24). Samm50 also plays a key role in regulating Pink1 degraded through direct interaction (25).

Although Samm50 is a key regulatory factor of Pink1 and might affect mitophagy, whether it regulate the Pink1-Parkin pathway and involve in cardiac hypertrophy remains poorly understood. In this study, we found that Samm50 was downregulated in the cardiac hypertrophy model. Samm50 knockdown or overexpression was confirmed to mitigate or aggravate Angiotensin II (Ang II) induced cardiomyocyte hypertrophy, respectively. Mechanistically, we found that Samm50 inhibit mitophagy through interacting with Pink1. Collectively, we propose that Samm50 regulates mitophagy in cardiomyocytes and mediates pathological hypertrophy.

## Materials and Methods

### Cell Culture and Treatment

Mouse neonatal ventricular cardiomyocytes were separated from 1–2-day-old neonatal C57BL/6J mice using enzymatic dissociation and cultured as previously described (26). Briefly, neonatal mice hearts were cut into small pieces and digested in 0.125 mg/ml trypsin (Gibco, #15090046) at 37°C. Then the supernatant was collected with complete Dulbecco's modified Eagle medium: Nutrient Mixture F-12 (DMEM/F12, Gibco, #8120319) and centrifuged at 600 g for 5 min. After repeating this cycle 6–8 times, the cell pellets were resuspended in DMEM/F12 containing 10% fetal bovine serum (FBS, Gibico, #10099133C), passed through a 100 mm cell strainer, and plated onto 10 cm dishes for 1.5 h at 37°C in 5% CO_2_. The supernatant was then obtained and plated on dishes for further experiments. For gene overexpression and knockdown studies, cardiomyocytes were infected with lentiviruses according to the manufacturer's protocol. Recombinant lentiviruses were designed and synthesized by Fubio Biological Technology. After lentiviral transduction for 72–96 h, cardiomyocytes were stimulated with 10^−6^ M Ang II (Sigma, #A9525) for the indicated time points.

HL-1 cardiac muscle cell line (HL-1 cell) was purchased from Sigma-Aldrich (#SCC065) and maintained in Claycomb medium (Sigma-Aldrich, #51800C) supplemented with 10% FBS and 2 mM L-glutamine. Plasmids were transfected according to the manufacturer's protocol. After starving for 24 h in serum-free Claycomb medium, the cells were treated with 10^−6^ M Ang II for 24 h and followed by co-immunoprecipitation (co-IP) experiments.

### Plasmids and Reagents

Pink1-Flag and Samm50-influenza hemagglutinin (HA) complementary DNA (cDNA) were cloned into the pcDNA3.1 vector. Short hairpin RNA (shRNA) against Samm50 and Pink1 were performed using the pLKO vector. The target sequences of shRNA oligonucleotides were listed in Supplementary Table 1. The Vps34 inhibitor was purchased from MCE (#HY-12794). Lipo8000 transfection reagent was purchased from Beyotime (#C0533).

### Transverse Aortic Constriction Model

C57BL/6J male mice (10–12 weeks) were subjected to transverse aortic constriction (TAC) to simulate the pressure overload model, and mice were sacrificed two weeks after surgery. After opening the chest cavity and separating the aortic arch, a 27-gauge needle was placed on the aorta between the left common carotid artery and the innominate artery, followed by ligation with 6-0 silk. Then, the needle was removed to generate aortic constriction. The Sham group mice underwent an identical surgery apart from the ligation. The animal study was reviewed and approved by the Animal Care and Use Committee of Zhongshan Hospital, Fudan University.

### RNA Isolation and Quantitative PCR Analysis

Total RNA was extracted with the TRIzol reagent (Ambion, #257401). cDNA was generated using PrimeScript^TM^ RT Reagent Kit with gDNA Eraser (Takara, #RR047A) following the manufacturer's instructions. Quantitative real-time polymerase chain reaction (qRT-PCR) was performed by ChamQ Universal SYBR qPCR Master Mix (Vazyme, #Q711-02) on a Bio-Rad IQ5 multicolor detection system. The program was as follows: 5 min at 95°C followed by 40 cycles of 20 s at 95°C and 30 s at 60°C. The results were analyzed using the 2^−Δ*ΔCt*^ method (27). The primer sequences were listed in Supplementary Table 2.

### Immunoblot and Co-immunoprecipitation Assay

Proteins were obtained from tissues and cells lysed in lysis buffer (Beyotime, #P0013B). Then, samples were separated by 10–15% SDS-polyacrylamide electrophoresis gel and transferred to PVDF membranes. After blocking in 5% BSA, blots were incubated with the primary antibodies at 4°C overnight, followed by incubation with peroxidase-conjugated rabbit secondary antibody (Thermo Fisher, #A0545, 1:5000) at room temperature for 1 h. The following antibodies were used in this study: anti-GAPDH (Proteintech, #HRP-60004, 1:10000), anti-LC3 (Cell Signaling Technology, #2775, 1:1000), anti-COX4 (Proteintech, #11242-1-AP, 1:1000), anti-Pink1 (Proteintech, #23274-1-AP, 1:1000), anti-Parkin (Proteintech, #14060-1-AP, 1:1000), anti-Samm50 (Abcam, #ab133709, 1:5000) and anti-TOM20 (Santa Cruz Biotechnology, #sc-11415, 1:1000).

For co-IP analysis, proteins were prepared with non-denaturing lysis buffer and incubated with primary antibodies against HA or isotype control immunoglobulin G (IgG) at 4°C overnight. Then, 50 μl magnetic beads (MCE, #HY-K0205) were added into the mixture and incubated for 4 h with rotation. After removing the surface attachments, samples were obtained from the bead-antibody complexes and subjected to immunoassay as above described. The following antibodies were used in this study: anti-IgG (Proteintech, #30000-0-AP, 1:50) and anti-HA (Proteintech, #51064-2-AP, 1:50).

### Immunofluorescence

Cells were fixed with 4% paraformaldehyde for 30 min, permeabilized with 0.1% Triton X-100 for 5 min, and blocked in 5% BSA for 1 h. Then cells were stained with the following primary antibodies overnight at 4°C: Cardiac troponin T (Abcam, #ab8295, 1:200), Samm50 (Proteintech, #28679-1-AP, 1:50), Pink1 (Proteintech, #23274-1-AP, 1:100) and VDAC1 (Santa Cruz Biotechnology, #sc-390996, 1:50). After washing with PBS 3 times, cells were incubated with specific secondary antibodies conjugated to Alexa Fluor for 1 h at 37°C. Five minutes after co-staining with 4',6-diamidino-2-phenylindole (DAPI, Invitrogen, #D1306), the cells were observed by a fluorescence microscope, and cardiomyocyte surface area was calculated by Image-Pro Plus software.

Heart sections were deparaffinized and rehydrated and then antigenically retrieved in sodium citrate buffer for 30 min. The following procedures were conducted according to the protocol described for cardiomyocytes. Co-localization was analyzed by Image-Pro Plus software.

### Measurement of Autophagy Flux

Adenovirus harboring tandem fluorescent mRFP-GFP-LC3 system (adenovirus-tf-LC3) was used to evaluate autophagy flux as previously described (28). Cardiomyocytes plated on coverslips were transfected with adenovirus-tf-LC3 at 10 MOI for 24 h and treated with Ang II for 6 h. Then cells were fixed with 4% paraformaldehyde, stained with DAPI, and observed under a fluorescence microscope. The number of GFP and mRFP dots were recorded by manual counting of fluorescent puncta from at least 50 cells. The number of DAPI-stained nuclei were recorded to represent the nuclear number.

### Statistics

All data were presented as mean ± s.e.m. All statistical results were analyzed using GraphPad Prism Software. Statistical analysis of two sets was performed by Student's t-test. Multiple comparisons were conducted by one-way analysis of variance with the Newman–Keuls test. A value of *P* < 0.05 was considered statistically significant.

## Results

### Samm50 Is Downregulated in Myocardial Hypertrophy

**Figure 1:**
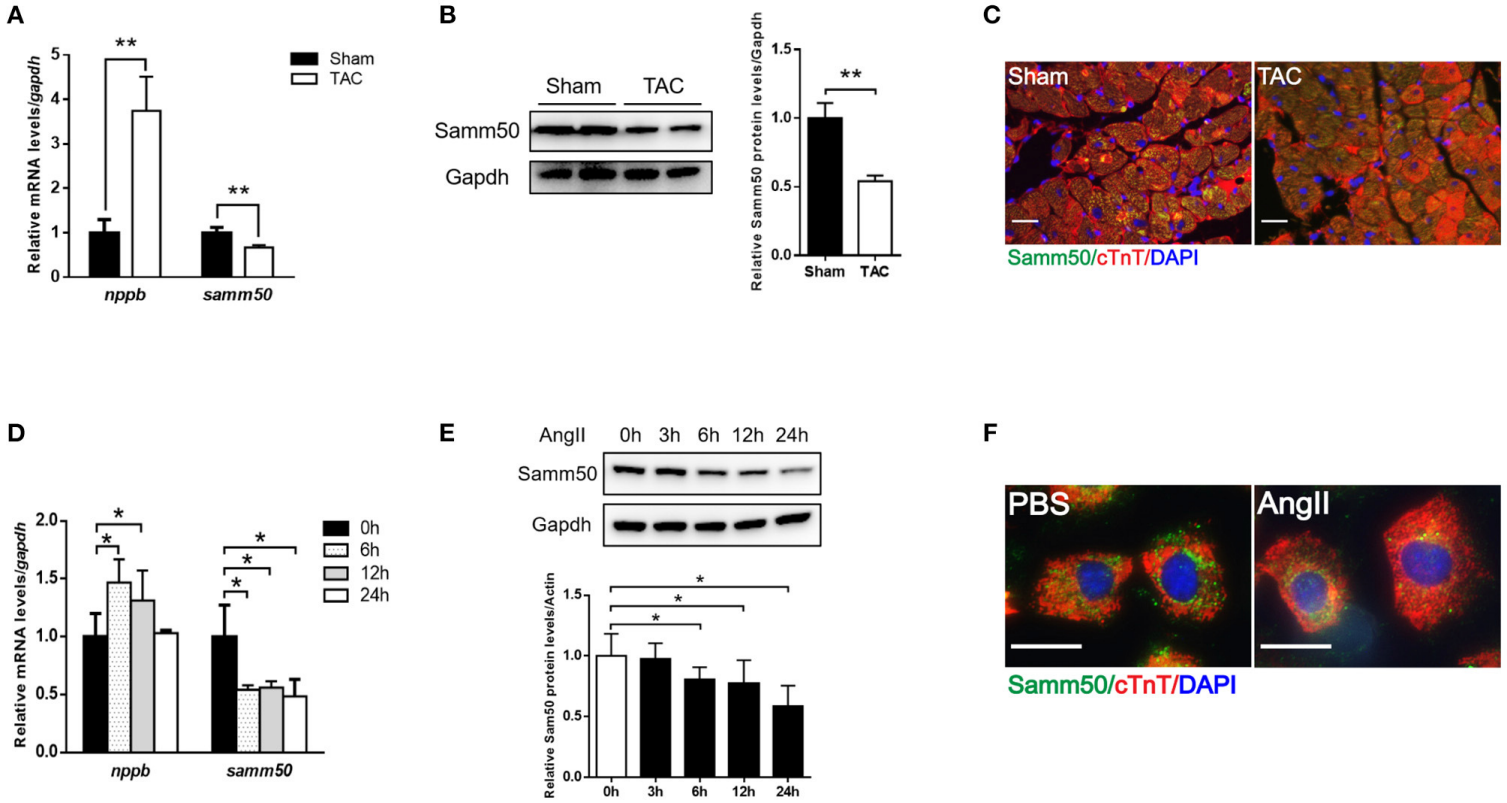
Samm50 is decreased in cardiomyocytes and ventricular tissues. **(A)** Quantification analysis of *samm50* and *nppb* expression in hearts of Sham and TAC mouse. **(B)** Representative western bolts (WBs) images and statistical results of *samm50* expression in cardiac tissues. **(C)** Immunofluorescence staining of *samm50* in mouse ventricular tissues of Sham and TAC group. Scale bars, 20 μm. *n* = 5 mice in each group. **(D,E)** Quantification (left) and WBs (right) of *samm50* expression in mouse neonatal cardiomyocytes treated with Ang II (10^−6^ M) for the indicated time points. **(F)** Immunofluorescence staining of endogenous *samm50* in mouse neonatal cardiomyocytes treated with PBS or Ang II. Scale bars, 20 μm. *n* = 3 independent experiments. ^**^P < 0.01 compared with Sham or PBS group; and ^*^P < 0.05 compared with Sham or PBS group. All data are presented as mean ± s.e.m.

To determine whether Samm50 expression is associated with cardiac hypertrophy, we performed the TAC mice model (Supplementary Figures 1A–D) and found the mRNA, and protein levels of Samm50 were remarkably decreased in response to pressure overload (Figures 1A,B). Parallelly, in the isolated mice neonatal cardiomyocytes, qRT-PCR and western bolt (WB) data showed that Samm50 expression levels were significantly reduced by Ang II stimulation (Figures 1D,E). Interestingly, Samm50 expression was unchanged in cardiac fibroblasts under Ang II treatment (Supplementary Figures 1E,F). The immunofluorescence staining also showed a consistent result of decreased Samm50 levels in TAC-induced hypertrophic hearts and Ang II-treated cardiomyocytes (Figures 1C,F). These results revealed that Samm50 was dramatically downregulated both *in vivo* and *in vitro* in response to hypertrophic stimuli, indicating a potential role of Samm50 in the regulation of cardiac hypertrophy.

### Overexpression of Samm50 Exacerbates Cardiomyocyte Hypertrophy and Inhibits Mitophagy

**Figure 2:**
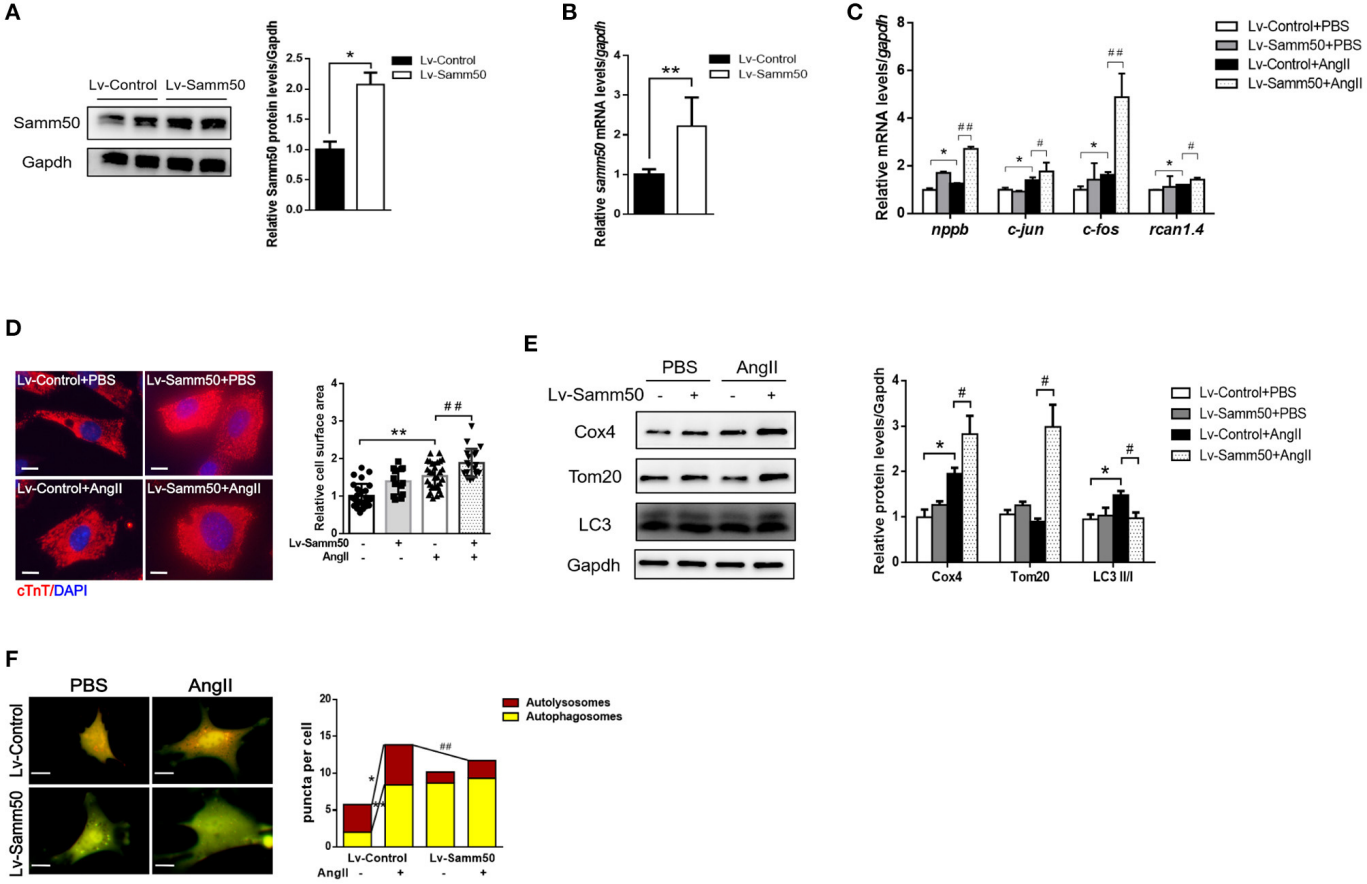
Samm50 exacerbates cardiomyocyte hypertrophy and inhibits mitophagy. **(A,B)** WB (left) and qRT-PCR (right) analysis of Samm50 overexpression levels in mouse neonatal cardiomyocytes. **(C)** Relative mRNA levels of hypertrophic markers gene expression in Lv-Control or Lv-Samm50 cardiomyocytes with Ang II treatment. Lv-Control, lentiviral pCDH-control and Lv-Samm50, lentivirus overexpressed Samm50. **(D)** Representative images and calculated cell surface area of cardiomyocytes infected with indicated lentivirus in response to Ang II stimulation. Scale bars, 10 μm. **(E)** Immunoblotting analysis of indicated protein levels in Lv-Control or Lv-Samm50 cardiomyocytes treated with PBS or Ang II. **(F)** Representative images of fluorescent LC3 puncta and statistical analysis results, and autolysosomes per cell. *n* = 6 for each experiment group. ^*^P < 0.05 compared with the control group; ^**^P < 0.01 compared with the control group; ^#^*P* < 0.05 compared with Ang II group; and ^##^*P* < 0.01 compared with Ang II group. All data are presented as mean ± s.e.m.

To investigate the potential effect of Samm50 on cardiomyocyte hypertrophy, we transfected lentivirus overexpressed Samm50 into isolated and cultured mice neonatal cardiomyocytes and verified Samm50 expression by WB and qRT-PCR analysis (Figures 2A,B). After Ang II administration, the expressions of hypertrophic markers such as *nppb* (natriuretic peptide B), *c-jun* (jun proto-oncogene), *c-fos* (Fos proto-oncogene), and *rcan1.4* (regulator of calcineurin 1, transcript 4) were obviously elevated in the Samm50-overexpressed group compared with the control group (Figure 2C). Furthermore, cardiomyocytes were incubated with cardiac troponin T to evaluate the cardiomyocyte surface area. As shown in Figure 2D, Samm50 overexpression markedly enlarged the cardiomyocyte size in response to Ang II treatment. These data indicated that Samm50 exacerbates cardiac hypertrophy.

Mitochondrial dynamics are critical to maintain cardiomyocyte function, especially under pathological stress. Given that Samm50 is associated with mitochondrial homeostasis and mitophagy (25), we explored whether Samm50 aggravates cardiac hypertrophy by affecting mitochondria function. We first evaluated mitochondrial biogenesis by detecting the expression of mitochondrial *NADH dehydrogenase 1* (*mtND1*). The results showed that *mtND1* was not altered in response to Samm50 overexpression (Supplementary Figure 1G). Then, autophagy markers and mitochondria protein levels were assessed by immunoblotting. Compared with the control group, overexpression of Samm50 displayed decreased LC3-II/LC3-I ratio and increased levels of TOM20 and COX4, indicating inhibition of mitophagy in cardiomyocytes (Figure 2E). Besides, the adenovirus-tf-LC3 was generated to evaluate autophagic flux. We monitored the number of red and green puncta by fluorescence microscope. The red puncta overlapped with the green ones are indicators of autophagosomes, while the free red puncta represent autolysosomes (29). As shown in Figure 2F, the yellow and red puncta were both increased in Ang II-treated cardiomyocytes, whereas Samm50 overexpression evidently decreased the number of red puncta, and most of them overlaid with the green ones in merged images, indicating Samm50 inhibits autophagic flux. Overall, these data demonstrated that Samm50 exacerbates Ang II-induced cardiomyocyte hypertrophy and inhibits mitophagy.

### Samm50 Depletion Attenuates Cardiomyocyte Hypertrophy by Promoting Mitophagy

**Figure 3:**
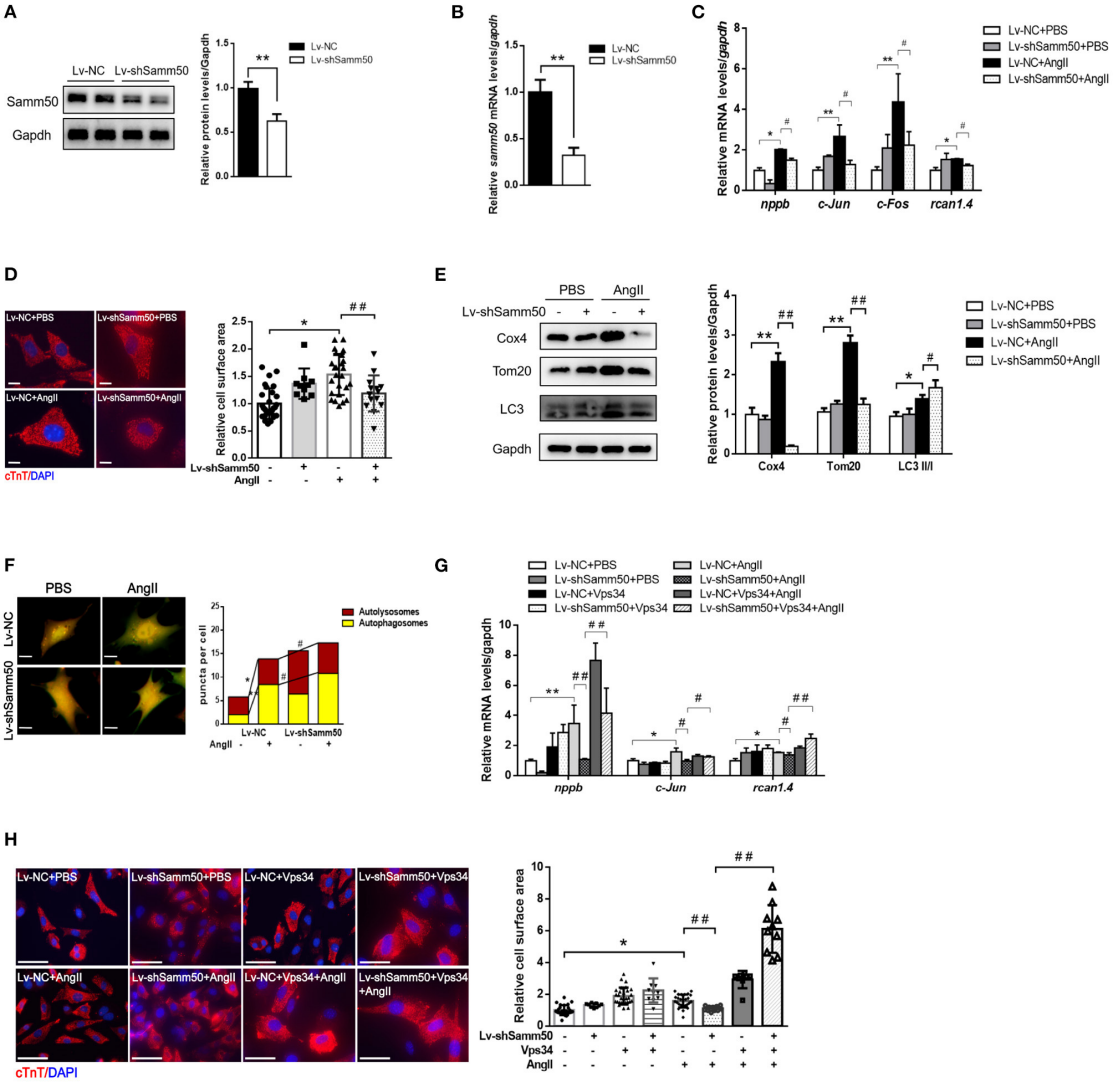
Samm50 deficiency alleviated cardiomyocytes hypertrophy by promoting mitophagy. **(A,B)** WB and qRT-PCR analysis of Samm50 knockdown efficiency in mouse neonatal cardiomyocytes. **(C)** Relative mRNA levels of hypertrophic marker gene expression in Lv-NC or Lv-shSamm50 cardiomyocytes with Ang II treatment. Lv-NC, lentivirus with pLKO control and Lv-shSamm50, lentiviral-delivered shRNA against Samm50. **(D)** Representative images and calculated cell surface area of cardiomyocytes with Ang II treatment. Scale bars, 10 μm. **(E)** Immunoblotting and statistical analysis of mitophagy protein levels in Lv-NC or Lv-shSamm50 cardiomyocytes treated with PBS or Ang II. **(F)** Representative images of fluorescent LC3 puncta and analysis results. Scale bars, 10 μm. **(G)** Quantification of hypertrophic markers expression in cardiomyocytes infected with indicated lentivirus and treated with or without Vps34 inhibitor prior to Ang II stimulation. **(H)** Representative images and quantitative results of cell surface area in response to Ang II. Scale bars, 50 μm. *n* = 6 for each experiment group. ^*^P < 0.05 compared with the control group; ^**^P < 0.01 compared with the control group; ^#^*P* < 0.05 compared with Ang II group; and ^##^*P* < 0.01 compared with Ang II group. All data are presented as mean ± s.e.m.

To further verify the effects of Samm50 on cardiac hypertrophy and heart failure, we performed lentivirus-delivered shRNA to ablate *samm50* expression. The efficacy of *samm50* depletion was confirmed by WB and qRT-PCR analysis, respectively (Figures 3A,B). In accordance with the results observed in Samm50 overexpression, Samm50 deficiency remarkably reduced the expression of hypertrophic markers and ameliorated cardiomyocyte size compared with the control group, as evidenced by qRT-PCR and immunofluorescence (Figures 3C,D). Knockdown of Samm50 also exhibited increased mitophagy, manifested by an increase in LC3-II/LC3-I ratio and a decrease in TOM20 and COX4. Furthermore, in response to Ang II treatment, Samm50 ablation caused a significant accumulation of red puncta, which was greater than that in yellow puncta, indicating Samm50 increases autophagosomes more than autophagosomes and thus stimulates autophagic flux (Figure 3F). These results suggested that Samm50 deficiency ameliorates cardiomyocyte hypertrophy and enhanced mitophagy.

To evaluate the role of mitophagy in Samm50-mediated cardiac hypertrophy, we introduced an autophagy inhibitor into the Samm50-depleted cardiomyocyte prior to Ang II stimulation. Notably, the protective effect of Samm50-deficiency on cardiomyocyte hypertrophy was largely diminished in the presence of autophagy inhibitors, as demonstrated by the increases of hypertrophic markers and cardiomyocyte size (Figures 3G,H), further supporting the idea that mitophagy is required for Samm50-mediated cardiomyocyte hypertrophy.

### Samm50 Regulates Pink1-Parkin-Mediated Mitophagy in Myocardial Hypertrophy

**Figure 4:**
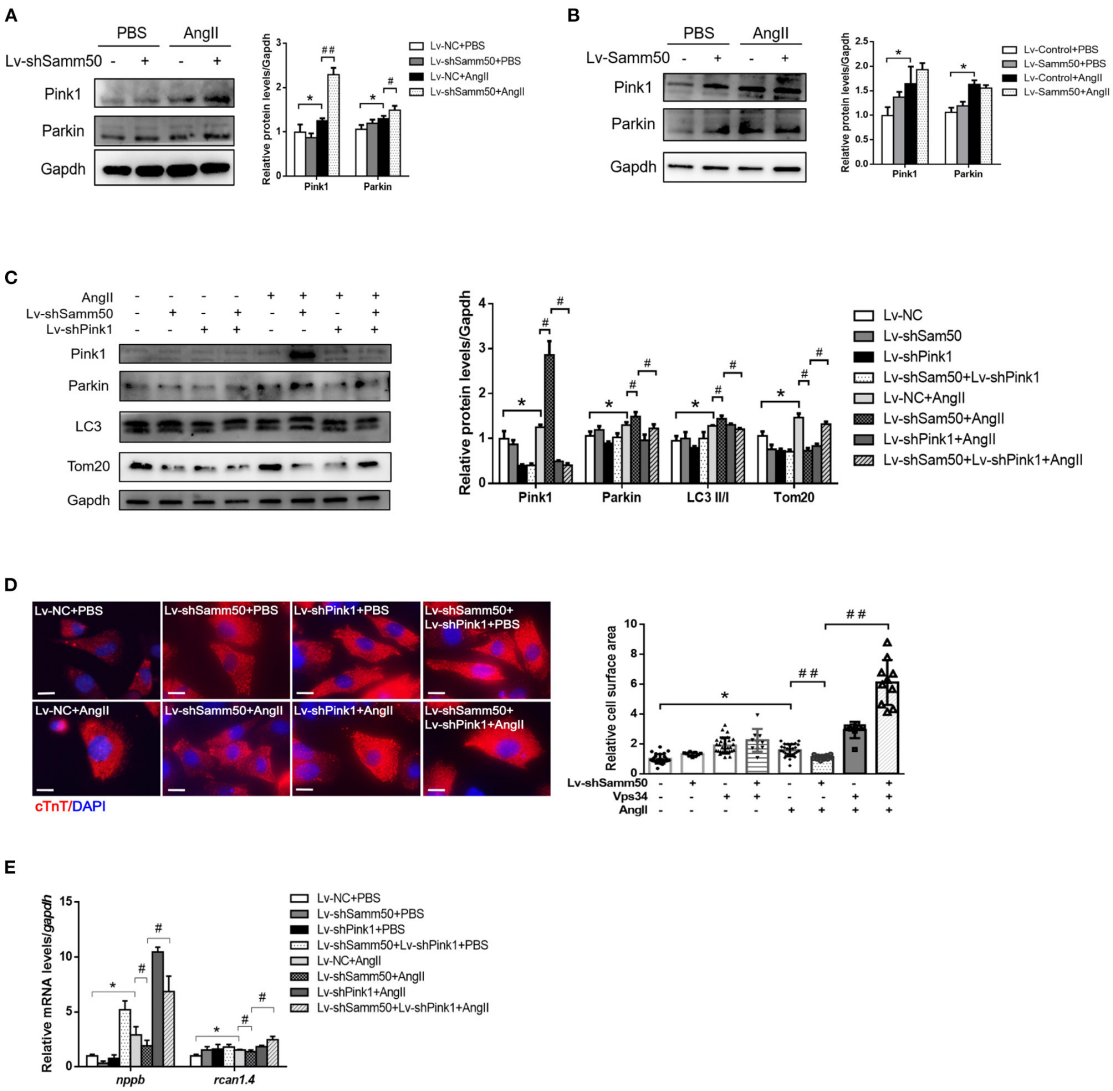
Samm50 regulates Pink1-Parkin-mediated cardiomyocyte mitophagy. **(A)** Cardiomyocytes transduced with Lv-NC or Lv-shSamm50 lentivirus were treated with Ang II and subjected to immunoblot analysis. **(B)** WB analysis of indicated proteins in Lv-control or Lv-Samm50 cardiomyocytes in response to Ang II. **(C)** Immunoblotting analysis for indicated proteins of control and Samm50-deficient cardiomyocytes infected with control or shPink1 lentiviral particles **(D,E)** Cell surface area and quantification of hypertrophic markers expression in cardiomyocytes infected with indicated lentivirus with Ang II stimulation. Scale bars, 10 μm. *P < 0.05 compared with the control group; ^#^*P* < 0.05 compared with Ang II group; ^##^*P* < 0.01 compared with Ang II group. All data are presented as mean ± s.e.m.

The previous study has suggested that Samm50-mediated mitophagy is dependent on the Pink1-Parkin pathway under cancer conditions (25). To further elucidate the underlying mechanism of Samm50 in the regulation of myocardial hypertrophy, we first detected the expression of Pink1 and Parkin in response to Ang II stimulation. As seen in Figure 4A, knockdown of Samm50 caused accumulation of Pink1 and Parkin, indicating the activation of mitophagy in cardiomyocytes. However, Pink1 and Parkin expression had no obvious difference between Samm50 overexpression and control group (Figure 4B). To evaluate whether Pink1 is required for Samm50-mediated cardiac hypertrophy, we interfered with the expression of Pink1 in the absence of Samm50. Our data revealed that Samm50 depletion-trigged activation of mitophagy was blunted in cardiomyocytes with simultaneous Pink1 knockout, as indicated by decreased LC3-II/LC3-I ratio and increased protein level of TOM20 and COX4 (Figure 4C). Consistent with immunoblotting results, the protective effect of Samm50 deficiency in response to Ang II induction was blocked by Pink1 knockdown, which was manifested by enhanced hypertrophic markers and cardiomyocyte enlargement (Figures 4D,E).

**Figure 5:**
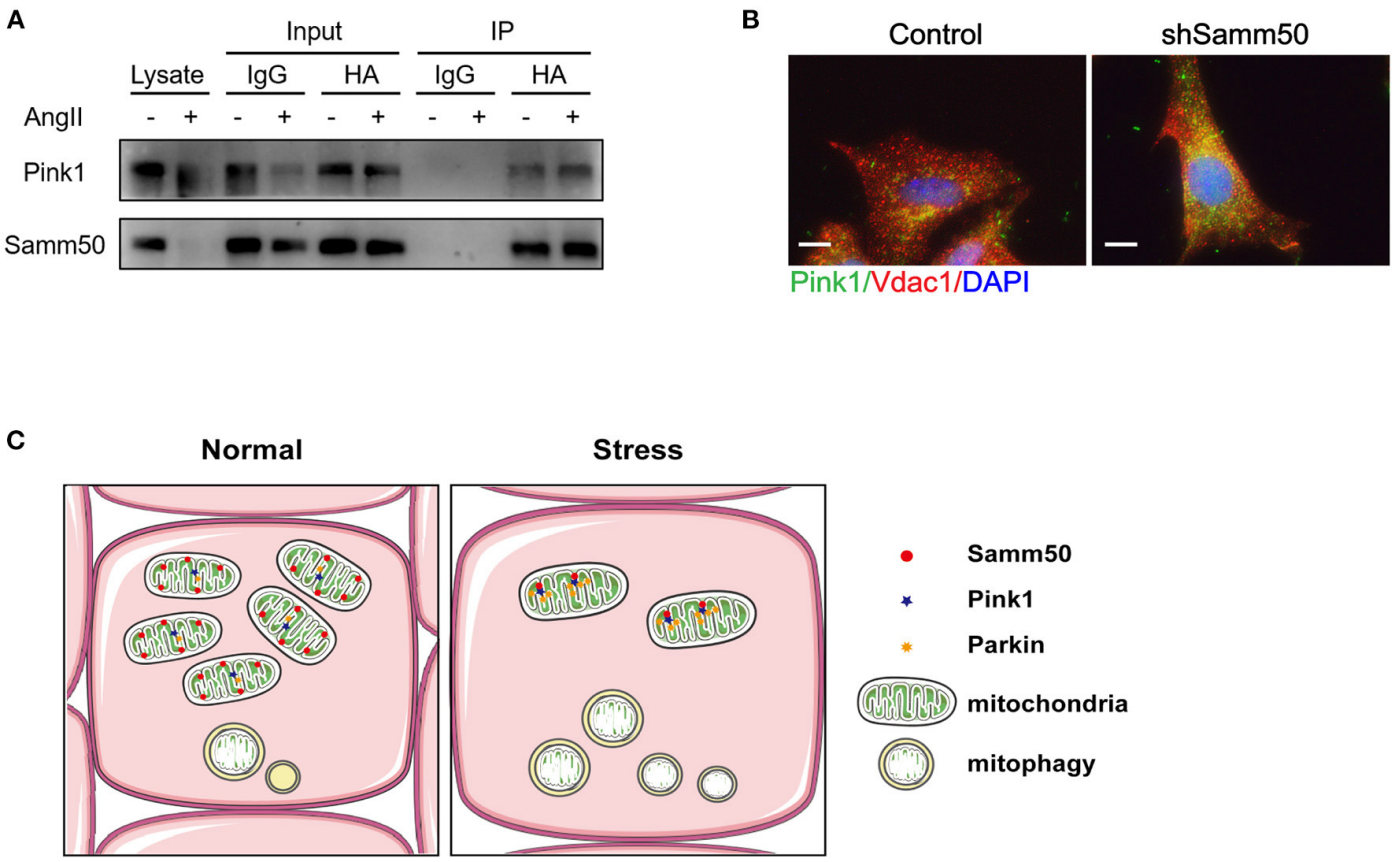
Samm50 interacts with Pink1 to mediate cardiac hypertrophy. **(A)** Co-immunoprecipitation analysis of Samm50 and Pink1 in HL-1 cells. **(B)** Representative immunofluorescence images of Pink1 and VDAC1 co-localization in mouse neonatal cardiomyocytes infected with control or shSamm50 lentiviral particles in response to Ang II. Scale bars, 10 μm. *n* = 6 for each experiment group. **(C)** The model of Samm50 regulates cardiac hypertrophy. Under cardiac hypertrophy, Samm50 interacts with Pink1 resulted in Pink1 accumulation, Parkin recruitment, and mitophagy.

Based on Samm50 interaction with Pink1 and regulation of its stability (25), we transfected HL-1 cells with HA-tagged Samm50 and Flag-tagged Pink1, followed by Ang II treatment and co-IP analysis to explore whether their physical interaction changes under Ang II stimulation. The co-IP of Samm50 by HA antibody exhibited an interaction between Samm50 and Pink1 in HL-1 cells, while there was no significant difference in the level of Pink1 detected in the absence or presence of Ang II, indicating the interaction between Samm50 and Pink1 was not influenced by Ang II stimulation (Figure 5A).

Pink1 and Parkin were recruited during mitophagy and accumulated in mitochondria (16). To further verify the activation of the Pink1/Parkin pathway in Samm50-mediated hypertrophy, we utilized the immunofluorescent staining to visualize the subcellular localization of Parkin and mitochondria in Samm50-deficient cardiomyocytes. The results showed that Parkin was predominantly stained with mitochondria marker VDAC1 (Figure 5B), indicating the accumulation of Parkin in mitochondria. Overall, these results demonstrate that Samm50 regulates cardiomyocyte hypertrophy in a Pink1/Parkin-dependent manner.

## Discussion

Cardiac hypertrophy is one of the primary reasons for heart failure. Pathological gene reprogramming could develop cardiac hypertrophy. This process is governed by multiple factors that could coordinate cardiac remodeling or against remodeling. Here, we identified a member of mitochondrial SAM complex, Samm50, which positively regulates cardiac hypertrophy involving mitophagy. We first observed the downregulation of Samm50 expression in the TAC model and Ang II-treated cardiomyocytes. Using gain- and loss-of-function approaches, we determined the potential role of Samm50 on pathological cardiac hypertrophy. Our results showed that Samm50 exacerbates cardiac hypertrophy by inhibiting mitophagy.

The more recent development of signaling effectors of cardiac hypertrophy is identified using genetically modified mouse models and primary cardiomyocytes. Mechanical stress, humoral stimuli, chromatin remodeling, inflammation, redox, and Ca^2+^ signaling were discovered to be strongly involved in cardiac hypertrophy (30). Furthermore, mitochondrial dysfunction has been indicated as a potential and important player in the development of cardiac hypertrophy (31). Mitophagy is essential to mitochondria homeostasis. However, few studies focus on the relationship between mitophagy and cardiac hypertrophy. One study showed that macrophage migration inhibitory factor significantly reduced pressure overload-induced cardiac hypertrophy by activating mitophagy and autophagy (32). Another one showed that the knockdown of lysocardiolipin acyltransferase 1 upregulates mitophagy to mitigate cardiac dysfunction associated with cardiac hypertrophy (33). Given that Samm50 was reported as a regulator of mitophagy and mitochondrial function, we first explored the involvements of mitochondria biogenesis in Samm50-mediated cardiomyocyte hypertrophy. Consistent with the previous study, the mitochondrial DNA copy number was not altered in these groups (Supplementary Figure 1G). We further observed that Samm50 depletion attenuates cardiomyocyte hypertrophy and promotes mitophagy (Figures 3C–F), indicating that Samm50 might inhibit mitophagy to exacerbate cardiac hypertrophy. Thus, we investigated the effect of autophagy inhibitors on the effect of Samm50 depletion in response to Ang II. The results revealed that the Vps34 inhibitor largely diminished the protective effect of Samm50 deficiency on Ang II-induced hypertrophy (Figures 3G,H), indicating the beneficial function of mitophagy in cardiac hypertrophy and that Samm50 regulates cardiomyocyte hypertrophy by inhibiting mitophagy. This result enlarged our understanding of mitophagy in cardiac hypertrophy.

Cardiac hypertrophy decompensation could be regulated by mitochondrial reprogramming. Samm50 is widely known as an essential protein of the mitochondrial outer membrane and has been verified to interact directly with the TOM complex to facilitate the biogenesis of ß-barrel protein. However, whether Samm50 participates in the cardiac hypertrophy process remains unclear. In our study, we found that the expression of Samm50 was significantly downregulated *in vivo* and *in vitro* cardiac hypertrophy model (Figures 1A–F), indicating that the changes of Samm50 maybe play a role in cardiac hypertrophy. To further clarify the relationship between Samm50 and hypertrophy, we conducted gain- and loss-of-function studies using lentivirus. We found that Samm50 overexpression aggravated the expression of hypertrophic markers and enlarged the cardiomyocyte size in response to Ang II (Figures 2C,D). Meanwhile, knockdown Samm50 could ameliorate cardiac hypertrophy (Figures 3C,D). Further understanding the mechanism by which Samm50 regulates mitophagy in cardiac hypertrophy is important for exploring the new strategies for treatment. Samm50 is a critical regulator of Pink1-Parkin-mediated or P62-mediated mitophagy (25, 34), whereas most mitophagy signals converge on the Pink1-Parkin-mediated pathway. Fenglei et al. revealed that Samm50 interacts with Pink1 and regulates its stability in HeLa cells (25). Agreed with the previous study, our results showed that Samm50 could directly interact with Pink1 in the cardiac hypertrophy model (Figure 5A). Besides, interference with Pink1 can eliminate the activation of mitophagy and the protective effect induced by Samm50 deficiency (Figures 4D,E). Collectively, we first discovered that Samm50-mediated mitophagy in cardiac hypertrophy was largely dependent on Pink1-Parkin signaling (Figure 5C). However, further experiments need to be performed to elaborate the mechanism involved in modulating Pink-Parkin by Samm50.

In conclusion, our present study shows Samm50 aggravates cardiac hypertrophy by regulating the Pink1-Parkin signaling and further clarifies the relationship between mitophagy and cardiac hypertrophy. These results may provide new insights into the treatment of cardiac hypertrophy.

## Data Availability Statement

The raw data supporting the conclusions of this article will be made available by the authors, without undue reservation.

## Ethics Statement

The animal study was reviewed and approved by the Animal Care and Use Committee of Zhongshan Hospital, Fudan University.

## Author Contributions

ZD, YZ, and RX designed the study. RX, ZD, LK, and SW predominantly performed experiments. RX, LK, CY, and YF analyzed the data. ZD and RX wrote the manuscript. YZ revised the manuscript. All authors read and approved the final version of the manuscript.

## Funding

This study was funded by the National Natural Science Foundation of China (Nos.: 81900245, 81730009, 81941002, and 81770395) and Science and Technology Commission of Shanghai Municipality (19YF1427900).

## Conflict of Interest

The authors declare that the research was conducted in the absence of any commercial or financial relationships that could be construed as a potential conflict of interest.

## Publisher's Note

All claims expressed in this article are solely those of the authors and do not necessarily represent those of their affiliated organizations, or those of the publisher, the editors and the reviewers. Any product that may be evaluated in this article, or claim that may be made by its manufacturer, is not guaranteed or endorsed by the publisher.
