# Supplementary figures and images for: Samm50 Promotes Hypertrophy by Regulating Pink1-Dependent Mitophagy Signaling in Neonatal Cardiomyocytes
Source: Front Cardiovasc Med. 2021 Sep 22;8:748156. doi: 10.3389/fcvm.2021.748156 (PMC8493082; doi:10.3389/fcvm.2021.748156)

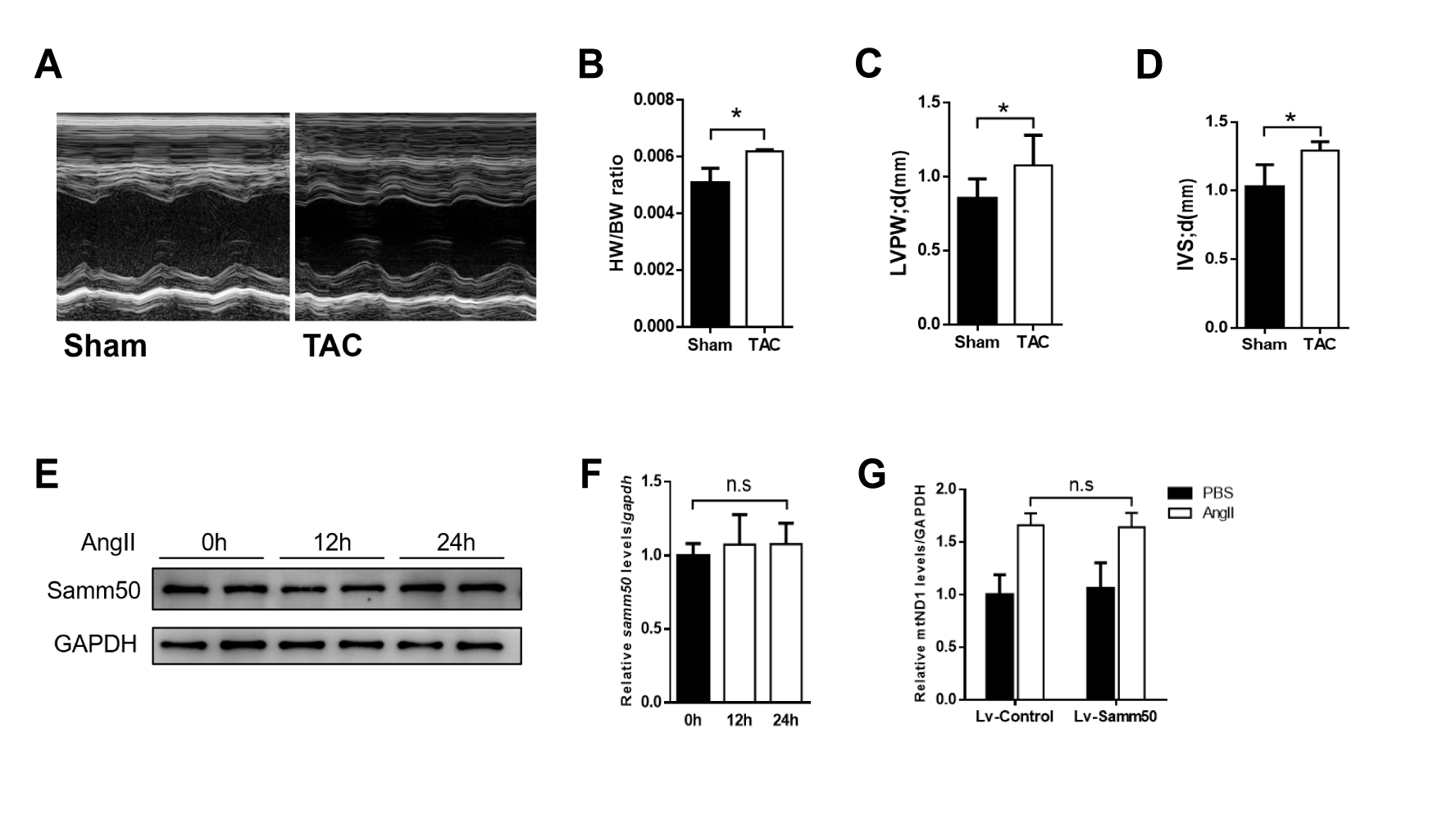

Supplement: Supplementary file 3 [file Image_1.TIF]
